# Prediction Technique and Measuring Device for Coupled Disturbance Forces from Large Equipment in the Spacecraft

**DOI:** 10.3390/s24041284

**Published:** 2024-02-17

**Authors:** Chengbo Zhou, Zhenbang Xu, Mingyi Xia

**Affiliations:** 1 Changchun Institute of Optics, Fine Mechanics and Physics, Chinese Academy of Sciences, Changchun 130033, China; zhouchengbo20@mails.ucas.ac.cn; 2 Center of Materials Science and Optoelectronics Engineering, University of Chinese Academy of Sciences, Beijing 100049, China

**Keywords:** micro-vibration, coupled disturbance force, prediction, flexible measuring platform, dynamic

## Abstract

To guarantee the accuracy of sophisticated equipment in spacecraft, it is essential to evaluate the dynamic forces of vibration sources. In contrast to conventional rigid-based measuring approaches, a method for predicting the interference of dynamic forces from large sources on spacecraft considering vibration coupling is proposed. In addition, a flexible-based dynamic force measuring platform capable of withstanding large masses and mounting large-volume vibration sources is designed. After that, the experiments for calibrating the platform and acquiring unknown terms in the derived theoretical models are detailed. The principle prototype is then manufactured for feasibility verification. It is demonstrated that despite the low fundamental frequency of the measuring platform of 242.8 Hz, the measurement error of the flexible measuring platform is less than 8% when the coupling is taken into account, which is 29% lower than that without coupling. Additionally, the prediction error of disturbance forces is within 17%. As a result, the accuracy of the proposed dynamic force measurement and prediction of large vibration sources considering coupling is substantially improved, providing a good reference for aerospace applications.

## 1. Introduction

A leading Chinese astronomy undertaking, the China Space Station Telescope (CSST), will be on par with the performance of the Hubble Telescope. For example, the CSST’s spectroscopic observations are designed to provide high-quality and slit-free spectra for hundreds of millions of stars and galaxies while enabling photometric surveys [1,2]. In addition, it has a wide field of view (1.1 square degrees) [3], which is much larger than that of the Hubble Telescope. Moreover, some WD binaries with high accretion rates can be detected by the CSST [4], which is of great interest to scientists. Because of this, the CSST places a great deal of demands on the installation environment, and even minor disturbance forces from vibration sources might have an impact on its operation [5,6]. This requires ground-based measurements of the disturbance forces generated by the sources and the use of the data to assess the effects of these forces on the telescope in the real environment. On the other hand, the CSST’s observation module has a diameter of over 1300 mm and a mass of over 300 kg [5]; hence, the ground-based measuring equipment needs to have a sizable mounting surface and a high load capacity. With the aforementioned measuring settings, there will inevitably be vibration coupling, which affects the measurement and prediction accuracy. Given the above, a dynamic force measuring platform with a larger mounting surface and load capacity is required. Additionally, a technique that takes coupling into account is needed to assess the impact of disturbance forces.

There are a number of works that can be used as a reference for the equipment and technology required. To improve the load capacity of force-measuring devices, piezoelectric ceramics have been employed as sensitive elements in earlier investigations [7,8,9,10,11]. Piezoelectric ceramics are highly suited for dynamic force measurements due to their good dynamic properties and substantially higher load capacity than strain gauge devices [12,13,14]. The load capacity of force measurement devices has also been increased by choosing four-point support structures. Li [15], for instance, suggested a parallel six-dimensional force sensor with a 10 kN load capacity, but this sensor can only be used to measure static forces. In order to mount several tons of vibration sources for dynamic force measurements, Xia [16] designed an eight-point-supported six-dimensional force measuring platform. However, its measurement principle is flawed. On the other hand, the Stewart structure [17,18,19] is rarely used in high-load force measuring equipment because of its low tangential load capacity, installation difficulties, and high processing costs. In order to overcome the problem of an insufficient mounting surface for the measuring device, Zhou [20] et al. proposed sensor-array and load-sharing force measuring platforms, but using them would make the measuring device very large and bulky if the diameter of the mounting surface was more than one meter. Most crucially, vibration coupling is rarely taken into consideration in the force measurements mentioned above, despite the fact that coupling can significantly affect the outcomes of dynamic measurements. Some researchers construct theoretical models of the vibration sources and utilize them to forecast the effects of dynamic forces in order to prevent vibration coupling during force measurements [21,22]. Xia [23], for example, improved the classical vibration model of the Momentum Wheel Assembly (MWA) to increase the accuracy of the model by accounting for structural coupling, but differences still exist between theoretical models and practical applications. Moreover, although there are some studies on the force measurement of small sources with consideration of vibration coupling [24,25], they are not directly applicable to the measurement of dynamic forces from large sources because small sources can be mounted directly on conventional rigid-based force measurement equipment. Further, Elias [26] discussed the prediction of the influence of the sources on the spacecraft and offered a specific testing strategy, but it was once again difficult to quantify some unknown terms in tests due to the enormous mass and volume of the sources. Nevertheless, these studies provide a good reference.

On the basis of the aforementioned, existing measurement and prediction methods have difficulty meeting the requirements presented. As a result, this paper proposes a novel measuring platform capable of mounting large volume and mass vibration sources, as well as a measurement model and a vibration prediction model that take vibration coupling into account. The mathematical models of measurement and prediction are described in Section 2. In Section 3, a flexible measuring platform is constructed and validated for the measurement of disturbance forces from large vibration sources. Section 4 carries out the crucial experiment design for measurement and prediction. The performance of the proposed flexible measuring platform and the viability of the mathematical models are next tested through experiments in Section 5. The findings of the research are compiled in the concluding section.

## 2. Models for Measurement and Prediction

### 2.1. Mathematical Model for Measuring Dynamic Forces

**Figure 1:**
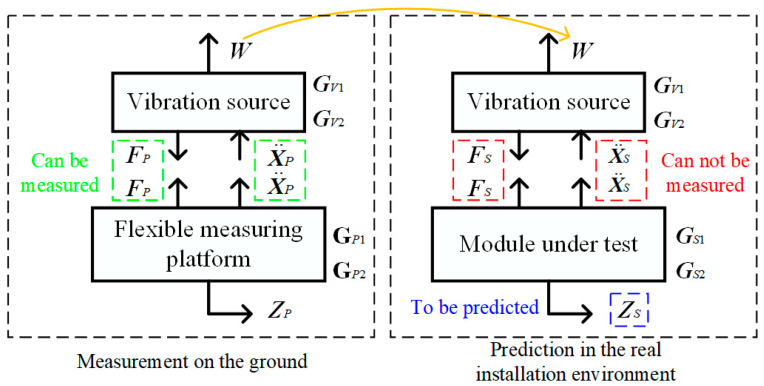
Mathematical models for the measurement of dynamic forces from a vibration source on the ground and disturbance prediction in the module considering dynamic coupling.

Before making a prediction about how the vibration source’s dynamic disturbance forces would affect the module in a realistic setting, it is crucial to measure the vibration source’s actual disturbance forces on the ground. The large vibration source will be coupled to the measurement platform, and the model for measuring disturbance forces while accounting for vibration coupling is shown in the left box of Figure 1, where ***F****_P_* and ${\ddot{X}}_{P}$ represent the forces and accelerations between the source and the flexible measuring platform, respectively, and these two terms can be measured as the source is mounted on the ground. The analysis in this section is based on a flexible measurement platform fixed to the ground and a vibration source mounted on top of the platform.

The equation of motion of the vibration source can be easily obtained from the existing kinematic theory [26]:

(1) $$F_{P(\omega )6\times 1}=G_{V1(\omega )6\times 6}W_{(\omega )6\times 1}+G_{V2(\omega )6\times 6}{\ddot{X}}_{P(\omega )6\times 1},$$

where ***W*** represents the disturbance forces/moments generated by the operating vibration source, and coefficients ***G****_V_*_1_ and ***G****_V_*_2_ are related to ***F****_P_*, $\ddot{X}P$, and ***W***. The measurement and prediction in this paper involve dynamic forces; hence, all of the derivations are based on frequency domain analysis, which necessitates first converting the time domain signals to the frequency domain. For the sake of brevity, (*ω*) is omitted in the subsequent paragraphs.

For the measuring platform, we can obtain similar expressions as shown in (2) and (3):

(2) $$Z_{P_{6\times 1}}=G_{{P1}_{6\times 6}}F_{P_{6\times 1}},$$

(3) $${\ddot{X}}_{P_{6\times 1}}=G_{{P2}_{6\times 6}}F_{P_{6\times 1}},$$

where ***G****_P_*_1_ relates ***F****_P_* to the performance of the platform ***Z****_P_*, and ***G****_P_*_2_ relates ***F****_P_* to $\ddot{X}P$. Substituting (3) into (1), (4) can be obtained:

(4) $$F_{P}=G_{V1}W+G_{V2}G_{P2}F_{P}$$

After rearranging (4), the expression for the relationship between ***W*** and ***F****_P_* can be found, as illustrated in (5):

(5) $$W=G_{V1}^{-1}(I-G_{V2}G_{P2})F_{P}=G_{P-V}F_{P}$$

where the matrix ***G****_V_*_1_ is invertible, and all subsequent matrices appearing in the article are also invertible.

Therefore, by measuring ***F**_P_*, it is possible to obtain the disturbance forces/moments of the vibration source ***W*** if the transfer function ***G****_P_*_−*V*_ is available. It should be noted that ***G****_V_*_2_ is typically obtained experimentally when ***W***(*ω*) is restricted to zero [26], as shown by (1). The convenient acquisition of ***G****_V_*_1_ requires $\ddot{X}P$ to be limited to zero, which becomes particularly difficult for large vibration sources because there is no rigid measuring platform with sufficiently large dimensions to guarantee that there are no accelerations at the mounting surface. Thus, the acquisition experiment for ***G****_V_*_1_ can become complicated.

### 2.2. Mathematical Model for Predicting Dynamic Forces

Next, as indicated by the yellow arrow in Figure 1, the disturbance forces/moments of the vibration source ***W*** measured on the ground can be used to predict the impact of the vibration source on the module. The model for the prediction considering vibration coupling is shown in the right box of Figure 1, where ***F****_S_* and ${\ddot{X}}_{S}$ represent the forces and accelerations between the vibration source and the module, respectively, and ***Z****_S_* denotes the performance of the module that is being predicted. The first two terms above cannot be measured due to the source being installed on the spaceship and the difficulty of installing sensors at their mounting interfaces. The analysis in this section is based on the fact that the module under test is fixed to the spacecraft, and the vibration source is mounted on top of the module.

Similar to (1)–(3), the equations of motion of the vibration source in the module can be expressed as follows:

(6) $$F_{S_{6\times 1}}=G_{{V1}_{6\times 6}}W_{6\times 1}+G_{{V2}_{6\times 6}}{\ddot{X}}_{S_{6\times 6}},$$

(7) $$Z_{S_{6\times 1}}=G_{{S1}_{6\times 6}}F_{S_{6\times 1}},$$

(8) $${\ddot{X}}_{S_{6\times 1}}=G_{{S2}_{6\times 6}}F_{S_{6\times 1}},$$

where ***G****_S_*_1_ relates ***F****_S_* to the performance of the module ***Z****_S_*, and ***G****_S_*_2_ relates ***F****_S_* to ${\ddot{X}}_{S}$. Taking (8) into (6) gives

(9) $$F_{S}=G_{V1}W+G_{V2}G_{S2}F_{S}$$

Rectifying (9) yields

(10) $$F_{S}={\left(I-G_{V2}G_{S2}\right)}^{-1}G_{V1}W$$

Combining (5), (7), and (10), the expression for the relationship between the performance of the module ***Z****_S_* and ***F****_P_* (***F****_P_* can be measured) can be obtained as follows:

(11) $$\begin{array}{ll} Z_{S} & =G_{S1}{\left(I-G_{V2}G_{S2}\right)}^{-1}G_{V1}W=G_{S1}{\left(I-G_{DV2}G_{S2}\right)}^{-1}G_{V1}G_{P-V}F_{P}\\ & =G_{S1}{\left(I-G_{V2}G_{S2}\right)}^{-1}G_{V1}G_{V1}^{-1}(I-G_{V2}G_{P2})F_{P}=G_{S1}{\left(I-G_{V2}G_{S2}\right)}^{-1}(I-G_{V2}G_{P2})F_{P} \end{array}$$

Equation (11) can be expressed as follows:

(12) $$Z_{S}=G_{P-S}F_{P}$$

(13) $$G_{P-S}=G_{S1}{\left(I-G_{V2}G_{S2}\right)}^{-1}(I-G_{V2}G_{P2})$$

Equations (12) and (13) illustrate that the performance of the module ***Z****_S_* can be predicted from the six-dimensional forces/moments ***F****_P_* between the vibration source and the measuring platform on the ground if the transfer function ***G****_P_*_−*S*_ is available. It is notable that the coefficient ***G****_V_*_1_ in the transfer function ***G****_P_*_−*V*_ is offset in the derivation of the prediction model, as shown in (11), which reduces the complexity of the experiment and is desirable to happen.

## 3. Structural Design of the Flexible Measuring Platform

The methods of measurement and prediction are derived in the previous section, and a measurement device is also required.

### 3.1. Fundamental Structure

**Figure 2:**
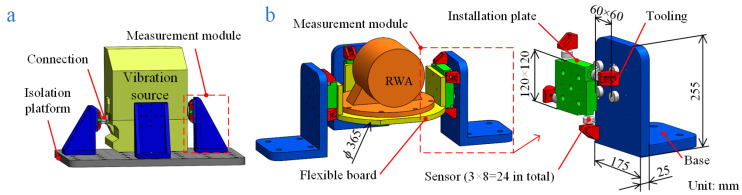
Schematic diagram of the platform structure: (**a**) structure of the platform for practical application; (**b**) structure of the designed flexible measuring platform for tests.

The vibration source shown in Figure 2a is the CSST’s sky inspection optics module, which has an outside envelope with a diameter of more than 1300 mm. Even Kistler, the best force measurement company in the world market, with its largest force measuring platform only being 400 × 400 mm in size, does not have a measurement device that can be mounted on the ground and measure the disturbance forces from this scale module. Moreover, their platforms only measure in the time domain without taking into account the coupling of vibrations.

Therefore, a brand new force measuring platform is proposed with coupling under consideration, as shown in Figure 2a. The measurement modules and the vibration source mounting interfaces are connected by connections. This measurement method significantly increases the upper limit of the volume of the installed vibration source. The output data from the measuring platform can be used for disturbance force measurement and prediction.

The principle prototype of the flexible measuring platform, depicted in Figure 2b, is designed for testing and simulation, which makes it easier to verify the theories in the previous section. The reaction wheel assembly (RWA) is used as the vibration source, and the structure of the measurement module is basically the same as that in Figure 2a. A flexible board is designed, and the stiffness of the base is reduced to make the principle prototype also coupled to the vibration source for verification purposes. The measuring platforms for the engineering application as well as the prototype use three measuring modules, each of which includes an installation plate, a base, four toolings, and eight piezoelectric force sensors, as illustrated in Table 1 (No. 1). Four piezoelectric force sensors are distributed in parallel between the bottom surface of the installation plate and the base. The other four sensors are fixed to the four sides of the installation plate using tooling that is mounted on the base. In order to increase the load capacity of the measuring platform, redundant sensors are linked, totaling 24 sensors in operation.

### 3.2. Structural Finite Element Analysis

**Figure 3:**
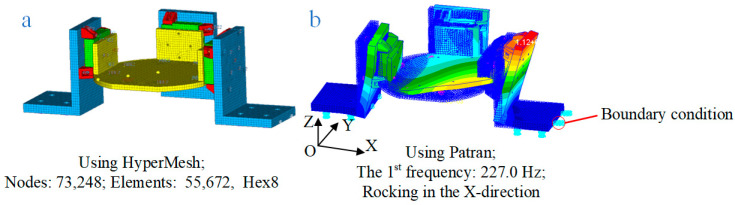
Finite element analysis of the flexible measuring platform: (**a**) the meshed model; (**b**) the platform’s first order of frequency.

To ensure that the designed flexible measuring platform couples with the RWA in the frequency range of interest (3–300 Hz, requirements encountered in aerospace engineering), finite element analyses (FEA) should be performed. The material properties of the platform during simulation are shown in Table 2. The mesh model of the platform adopting the element type of Hex8 with good computational efficiency and accuracy is shown in Figure 3a, and there are 73,248 nodes and 55,672 elements. The bottom of the platform is fixed, and its frequency response is simulated modally.

The simulation results are displayed in Figure 3b (redder colors represent larger displacements), where the fundamental frequency of the measuring platform is 227.0 Hz, which is in the range of 3–300 Hz. Therefore, the designed flexible measuring platform and the RWA are coupled, which is consistent with the actual measurement situation.

## 4. Experimental Designs

The purpose of the experimental designs is to calibrate the flexible measuring platform and identify the unknown terms in the transfer functions **G***_P_*_−*V*_ and **G***_P_*_−*S*_. Then, the platform can accurately measure ***F****_P_*, thus making (5) and (12) practically usable for measurement and prediction.

### 4.1. Experimental Design for the Calibration

The measurement of the flexible measuring platform is carried out according to (14):

(14) $$F_{6\times 1}=D_{6\times n}V_{n\times 1},$$

where ***F*** is the calculated six-dimensional forces/moments, ***V*** is the output data from *n* of the 24 sensors, and ***D*** is the calibration matrix to be obtained.

The calibration of the flexible measuring platform is carried out in the frequency domain in a specialized calibration laboratory. By applying the known forces to the known points and gathering the *n* outputs from 24 force sensors, calibration can be carried out.

In the calibration experiment, ***F****_c_* is the known six-dimensional forces/moments (subscript *c* means calibration), and ***V****_c_* is the output voltage of the sensors through the amplifier. The equation can be obtained as follows:

(15) $$V_{c6\times 1}=E_{c6\times 6}F_{c6\times 1}$$

The coefficient matrix ***E****_c_* can be obtained using the generalized inverse of the matrix:

(16) $$E_{c}=V_{c}{F_{c}}^{T}{\left[F_{c}{F_{c}}^{T}\right]}^{-1}$$

(17) $$F_{c}’={\left[{E_{c}}^{T}E_{c}\right]}^{-1}E_{c}V_{c}$$

Substituting (16) into (15) yields (17).

At this point, (14) and (17) are comparable, and it becomes clear that [***E****_c_*^T^***E****_c_*]^−1^***E****_c_* is the required calibration matrix ***D***. In addition, a discussion of how to select *n* of the 24 sensors for calibration and measurement will give higher accuracy is described in [20].

**Figure 4:**
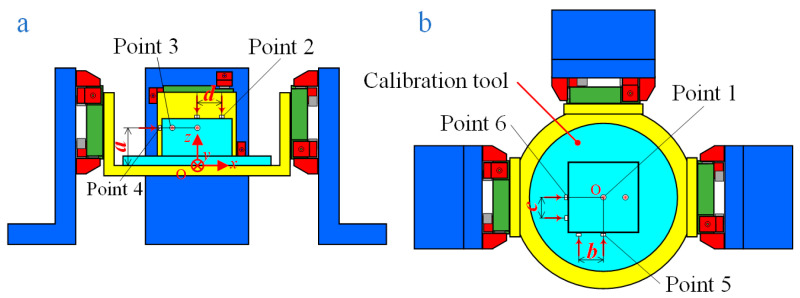
Platform calibration diagram: (**a**) front view; (**b**) top view.

The specific steps of the calibration experiment are designed next. As shown in Figure 4, the calibration tool is installed on the flexible board in order to input known six-dimensional forces/moments to the measuring platform. Six calibration points are selected on the calibration tool, and it is necessary to make sure that the input forces from the six points can completely cover the six-dimensional forces/moments in space after conversion to the center point O of the measuring platform. After that, using calibrating equipment [20] which is capable of applying forces perpendicular to the surfaces on which the calibration points are located, the forces are sequentially applied to each of the six points. It needs to be noted that the input forces ***F***_in_ should be converted into the forces ***F****_c_* with point O as a reference, according to (18):

(18) $$F_{c}(t)=CF_{\mathrm{in}}(t)=\left[\begin{array}{cccccc} 0 & 0 & 0 & 1 & 0 & 1\\ 0 & 0 & 1 & 0 & 1 & 0\\ -1 & -1 & 0 & 0 & 0 & 0\\ 0 & 0 & -a & 0 & -a & 0\\ 0 & d & 0 & a & 0 & a\\ 0 & 0 & -b & c & 0 & 0 \end{array}\right]\left[\begin{array}{cccccc} F_{1}(t) & 0 & 0 & 0 & 0 & 0\\ 0 & F_{2}(t) & 0 & 0 & 0 & 0\\ 0 & 0 & F_{3}(t) & 0 & 0 & 0\\ 0 & 0 & 0 & F_{4}(t) & 0 & 0\\ 0 & 0 & 0 & 0 & F_{5}(t) & 0\\ 0 & 0 & 0 & 0 & 0 & F_{6}(t) \end{array}\right],$$

where *F_i_*(*t*) is the input force at the *i*^th^ point at moment *t*, and ***C*** is the transform matrix, which can be obtained according to Figure 4.

The calibration matrix, as well as the optimal combination of *n* sensors used in the calibration and measurement of the platform, can be obtained through experiments.

### 4.2. Experimental Design for the Terms in Mathematical Models

The transfer functions ***G****_P_*_−*V*_ and ***G****_P_*_−*S*_ contain the unknown terms ***G****_V_*_1_, ***G****_V_*_2_, ***G****_P_*_2_, ***G****_S_*_1_, and ***G****_S_*_2_. If the first three terms are known, the measurement model is available, and if the last four terms are known, the prediction model can be applied.

Next, the acquisition of ***G****_V_*_2_ is emphatically analyzed.

Equation (1) shows that the acquisition of ***G****_V_*_2_ is straightforward when the vibration source is not operating (for instance, when the RWA stops rotating and the disturbance W vanishes):

(19) $$W=0,F_{P}=G_{V2}{\ddot{X}}_{P}$$

In this case, the term ***G****_V_*_2_ can be obtained by measuring the forces and accelerations on the mounting surface of the vibration source:

(20) $$G_{V2}=F_{P}{\ddot{X}}_{P^{-1}}$$

**Figure 5:**
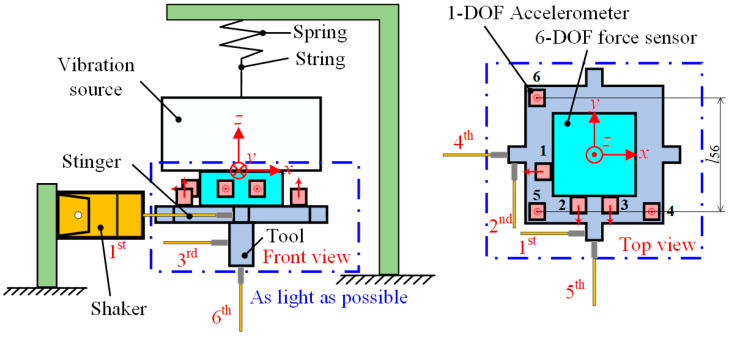
Schematic diagram of the Free-Free experiment.

The test here is carried out in a suspended manner, using boundary conditions where both displacement and force are unconstrained, called the Free-Free experiment. To make sure that the fundamental frequency of the vibration source is below 3 Hz at this time, the source is suspended using a string and spring, as shown in Figure 5. In order to input six-dimensional forces/moments to the mounting surface of the source, the tool shown in Figure 5 is installed. One 6-DOF force sensor and six 1-DOF acceleration sensors are installed in order to detect the forces and accelerations on the mounting surface of the vibration source.

The shaker is used to input white noise disturbances to the source through the stinger and tool. Subsequently, the corresponding forces and accelerations are generated on the mounting surface, and sufficient signals can be collected to derive the transfer function ***G****_V_*_2_, where the stinger serves to make the forces transfer from the shaker to the tool unidirectionally. As demonstrated in Figure 5, a total of six white noise disturbance forces are input at different points on the tool. Then, the outputs of the sensors are collected each time. The input forces at the six points transformed into the forces at the center of the source should fully encompass the six-dimensional forces/moments in space.

Therefore, the ***F****_P_* in (20) is as follows:

(21) $$F_{P}=\left[\begin{array}{cccccc} F_{1} & F_{2} & F_{3} & F_{4} & F_{5} & F_{6} \end{array}\right],$$

where ***F****_i_* (*i* = 1, 2, …, 6) is the six-dimensional force measured by the force sensor for the *i*^th^ time, which can be expressed as shown in (22):

(22) $$F_{i}={\left[\begin{array}{cccccc} F_{xi} & F_{yi} & F_{zi} & M_{xi} & M_{yi} & M_{zi} \end{array}\right]}^{T}$$

In addition, the ${\ddot{X}}_{P}$ in (20) can be expressed as follows:

(23) $${\ddot{X}}_{P}=\left[\begin{array}{cccccc} {\ddot{X}}_{1} & {\ddot{X}}_{2} & {\ddot{X}}_{3} & {\ddot{X}}_{4} & {\ddot{X}}_{5} & {\ddot{X}}_{6} \end{array}\right],$$

where ${\ddot{X}}_{i}$ (*i* = 1, 2, …, 6) is the six-dimensional acceleration measured by the acceleration sensors for the *i*^th^ time, which can be expressed as shown in (24):

(24) $${\ddot{X}}_{i}={\left[\begin{array}{cccccc} {\ddot{x}}_{i} & {\ddot{y}}_{i} & {\ddot{z}}_{i} & {\ddot{\theta }}_{xi} & {\ddot{\theta }}_{yi} & {\ddot{\theta }}_{zi} \end{array}\right]}^{T}$$

The six-dimensional accelerations in (24) are not obtained directly by the sensors and require the processing of the output signals based on the mounting configuration of the sensors in Figure 5, which is shown in (25):

(25) $$\left\{ \begin{array}{l} {\ddot{x}}_{i}=-a_{1i},{\ddot{y}}_{i}=-\frac{1}{2}(a_{2i}+a_{3i}),{\ddot{z}}_{i}=\frac{1}{3}(a_{4i}+a_{5i}+a_{6i})\\ {\ddot{\theta }}_{xi}=\frac{1}{l_{56}}(a_{6i}-a_{5i}),{\ddot{\theta }}_{yi}=\frac{1}{l_{45}}(a_{5i}-a_{4i}),{\ddot{\theta }}_{zi}=\frac{1}{l_{23}}(a_{2i}-a_{3i}) \end{array}\right.,$$

where *a_ji_* is the acceleration measured by the *j*^th^ acceleration sensor at the *i*^th^ time. Based on the above, ${\ddot{X}}_{i}$ can be expressed as (26).

(26) $${\ddot{X}}_{i}=\left[\begin{array}{cccccc} -1 & 0 & 0 & 0 & 0 & 0\\ 0 & -\frac{1}{2} & -\frac{1}{2} & 0 & 0 & 0\\ 0 & 0 & 0 & \frac{1}{3} & \frac{1}{3} & \frac{1}{3}\\ 0 & 0 & 0 & 0 & -\frac{1}{l_{56}} & \frac{1}{l_{56}}\\ 0 & 0 & 0 & -\frac{1}{l_{45}} & \frac{1}{l_{45}} & 0\\ 0 & \frac{1}{l_{23}} & -\frac{1}{l_{23}} & 0 & 0 & 0 \end{array}\right]\left[\begin{array}{c} a_{1i}\\ a_{2i}\\ a_{3i}\\ a_{4i}\\ a_{5i}\\ a_{6i} \end{array}\right]=T_{a}A_{i}$$

Thus, ***G****_V_*_2_ can be derived by substituting (21), (22), (23), and (26) into (20). Other unknown terms can be obtained in the same way as above.

**Figure 6:**
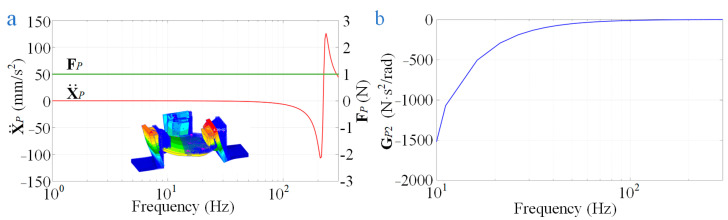
Diagram of the terms: (**a**) terms at position (1,1) in matrix ${\ddot{X}}_{P}$ and ***F****_P_*; (**b**) the term at position (3,4) in matrix ***G****_P_*_2_.

Finite element simulation is another method that can be used to obtain the unknown terms in addition to the experimental approach. Taking the unknown term ***G****_P_*_2_ in (3) as an example, by inputting a suitable dynamic force on the mounting surface of the flexible measuring platform, the displacement of its mounting surface can be obtained through the simulation of the Patran software, and its acceleration can be calculated from the displacement. The input dynamic forces ***F****_P_* and the accelerations of the mounting surface ${\ddot{X}}_{P}$ in (3) are both 6 × 6 vectors (input six single-dimensional forces), which makes ***G****_P_*_2_ a 6 × 6 vector. The terms at position (1,1) in matrix ${\ddot{X}}_{P}$ and ***F****_P_* and the term at position (3,4) in matrix ***G****_P_*_2_ are shown in Figure 6.

When the structure is simple, it is easier to obtain the unknown terms by simulation with satisfactory accuracy. Regarding the acquisition of the remaining items, they will not be repeated here, which can all be acquired by similar or appropriately extended experiments.

## 5. Experiment

The experimental prototype was built and used to validate the above theory and scheme. First, the flexible measuring platform was dynamically calibrated. The disturbance forces of RWA were then measured using both flexible and rigid measuring platforms, and the results of both were compared. Following that, the measurement results of RWA considering the vibration coupling can be obtained to confirm the feasibility of the measurement model for the flexible platform. Finally, the reliability of the prediction model was verified, and practical applications were carried out.

### 5.1. Calibration Experiments for the Flexible Measuring Platform

**Figure 7:**
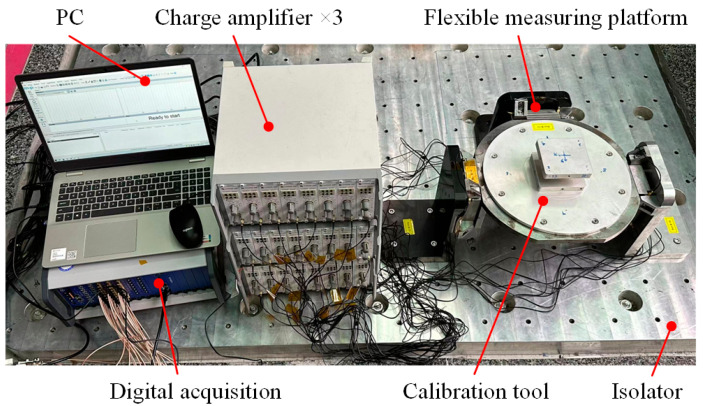
Calibration setup of the flexible measuring platform.

The calibration setup for the flexible measuring platform is shown in Figure 7, including the flexible measuring platform, the charge amplifier (CT5853), the digital acquisition (VRAI820), the calibration equipment [20] (homemade; not displayed; the force sensor used is shown in Table 1, No. 2), the calibration tool, and the PC.

**Figure 8:**
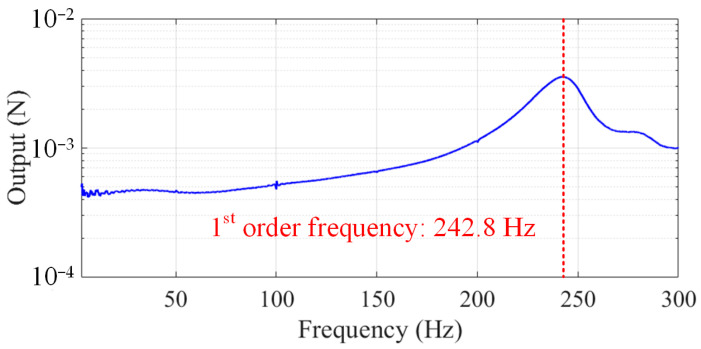
Fundamental frequency of the flexible measuring platform.

The fundamental frequency of the platform can first be obtained at 242.8 Hz using the experiment of hammering, as shown in Figure 8 (output of a sensor), which is consistent with the fundamental frequency of 227 Hz obtained from the simulation. After that, the calibration forces were input, and the outputs of the sensors were collected according to the above calibration experiment steps. By using D optimization, the optimal combination of sensors as well as the calibration matrix of the platform may be obtained.

**Figure 9:**
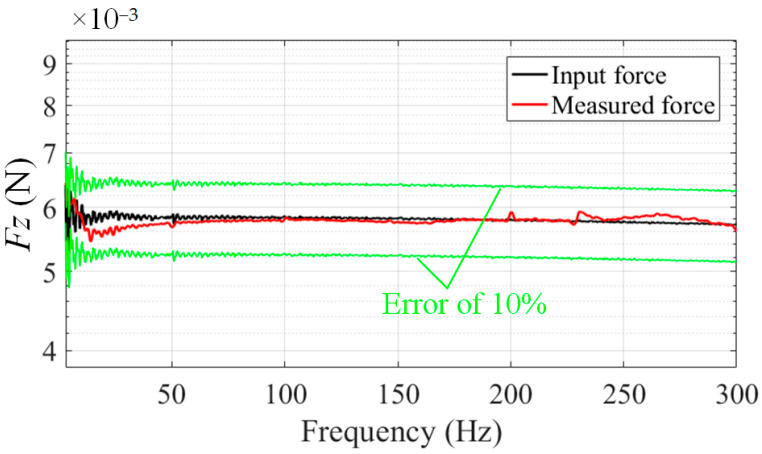
Measurement of impulsive force on the mounting surface of the flexible measuring platform.

After calibrating the flexible measuring platform, known impulsive forces were input into the calibration tool and measured using the flexible measuring platform. The result is shown in Figure 9 (only *F_z_* are displayed). It can be determined that the dynamic error of the mounting surface of the platform is within 1.5% after calibration.

### 5.2. Comparison of the Measurements of Platforms without Considering Coupling

**Figure 10:**
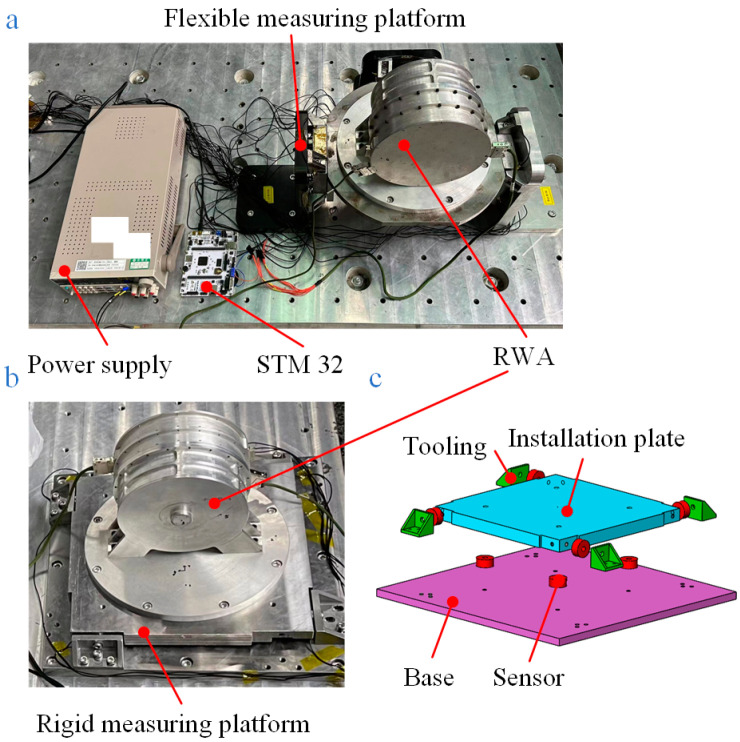
Physical view of the platforms used to measure the disturbance forces between RWA and their mounting surfaces: (**a**) flexible measuring platform; (**b**) physical drawing of the rigid measuring platform; (**c**) schematic structure of the rigid measuring platform.

**Figure 11:**
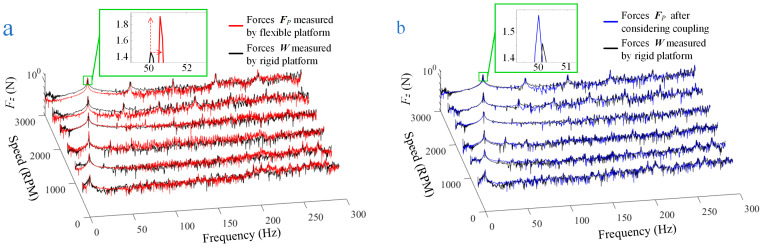
Comparison of results: (**a**) ***F****_P_* and ***W***; (**b**) the output of RWA measured using the flexible measuring platform considering vibration coupling and ***W***.

As shown in Figure 10a, the disturbance forces ***F****_P_* (the force on the z-axis is depicted, as is the article’s follow-up) between the RWA and its mounting surface were measured by the flexible platform, and the result is represented by the red line in Figure 11. To illustrate the difference between ***F****_P_* and the real disturbance force of RWA ***W***, ***W*** was measured using a rigid measuring platform shown in Figure 10b (precision error: <3%, the structure of which is shown schematically in Figure 10c), which is depicted as a black line in Figure 11.

According to the experimental findings, the flexible measuring platform’s measurement error in the entire frequency range is within 171% without taking vibration coupling into account, and the average amplitude error of the frequency points of interest is less than 37%. The expanded picture in Figure 11a also reveals that, in the situation of vibration coupling, both the frequency points characterizing the RWA and the corresponding amplitudes are amplified. Certainly, if the fundamental frequency of the measurement platform is set lower, the amplification will be more pronounced. In order to objectively represent the measurement accuracy of the platform, data for the key frequencies in Figure 11a are included in Table 3 (red line).

The vibration source used in this paper is quite small and can therefore be measured using a rigid platform for verification purposes. If the dimensions of the source are as huge as in practical applications, measurements can only be made using a flexible platform, and comparison is not available. This is exactly the purpose of the design of the principle prototype.

### 5.3. Measurement of the Flexible Platform Considering Coupling

Based on the measured disturbance force ***F****_P_* between the RWA and the mounting surface of the flexible platform, the disturbance output ***W*** of the RWA considering the vibration coupling can be calculated using the measurement model as shown in (5). The output of RWA measured with the flexible measuring platform considering vibration coupling (blue line) and the output of RWA measured with the rigid measuring platform (black line; as a reference) are shown in Figure 11b.

**Figure 12:**
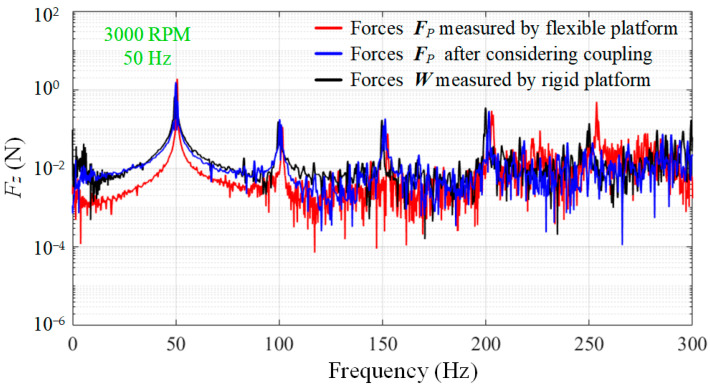
Comparison of measurement accuracy at 3000 RPM.

The experimental results indicate that, when coupling effects are taken into account, the measurement error of the flexible measurement platform is less than 85% across the entire frequency band (this cannot be used as a reliable reference for comparison due to interference from random noise and small amplitude), and the average error of the amplitude of the frequency points of interest is less than 8%, which is a significant improvement in accuracy. Further, the comparison in Figure 12 gives a better view of the enhanced accuracy of the measurements. Table 3 also contains quantitative data for the key frequencies covered in Figure 11b, from which we can see that not only the amplitude accuracy becomes higher, but also the frequency point accuracy is improved.

This cannot be compared to the results of others in terms of measurement accuracy because no one has ever developed a flexible measuring platform that is as adaptable for heavy loads. Nevertheless, the measurement precision is adequate.

### 5.4. Experiment for Prediction of Disturbance Forces Considering Coupling

**Figure 13:**
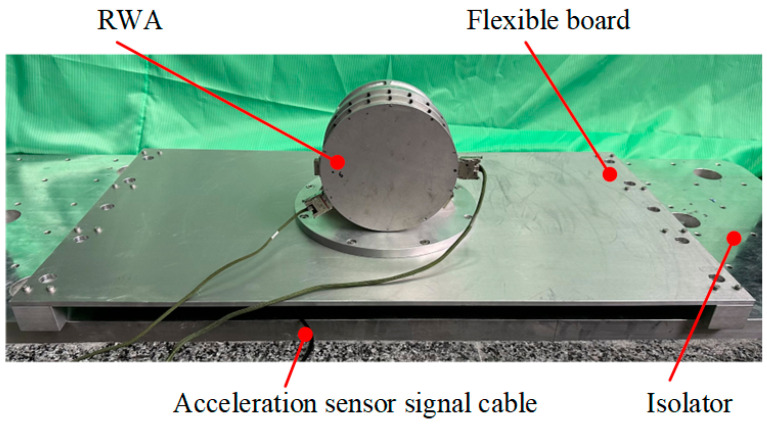
Predictive experimental installation configuration of RWA.

The impact of RWA in a simulated installation environment can be obtained by taking the ***F****_P_* obtained from the flexible platform in the previous section into the prediction model, as shown in (12). In order to facilitate the experimental verification, a simple flexible board, as illustrated in Figure 13, was chosen as the installation environment for the RWA, and the output at the center of the mass of the flexible board is to be predicted. Additionally, the acceleration sensors are installed at the center of the mass of the flexible board to measure and calculate the output.

**Figure 14:**
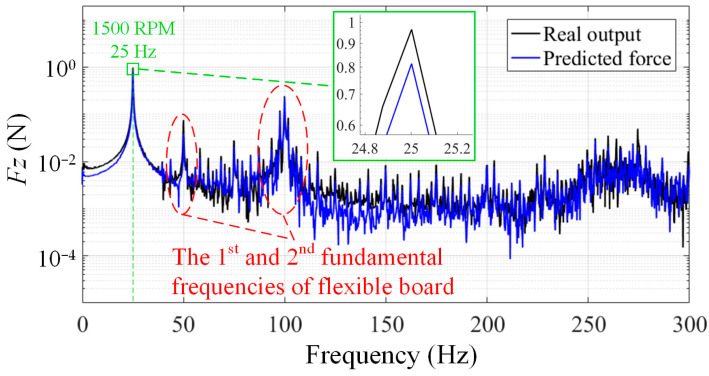
Comparison of the model-predicted disturbance force output at 1500 RPM and the real output measured at the center of the mass.

Figure 14 compares the predicted output of the disturbance forces using the proposed model with the real output measured at the center of mass. From the experimental results, the model’s prediction error at the frequency points of interest is within 17%, which is satisfactory. The frequencies in the red box in Figure 14 are from the flexible board itself, which should be predicted and have been predicted. Naturally, part of the reason for its good predictive results is the simplicity of the installation environment.

The experiment in Section 5.3 is intended to confirm the viability of the measurement model, and it is also necessary to obtain the term ***G****_V_*_1_, which is more challenging to obtain experimentally. In practice, however, tests identical to those in this section are sufficient to forecast the effects of dynamic disturbances on the module; obtaining ***G****_V_*_1_ is not necessary, thus streamlining the experimental procedure.

### 5.5. Application

**Figure 15:**
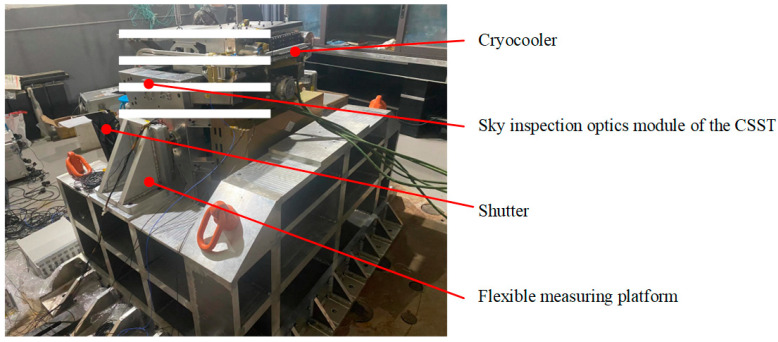
Application of the designed platform in practice.

The above-mentioned prediction model has been applied to the actual measurement, as shown in Figure 15, where the white mosaic covers the sky inspection optics module of the CSST for confidentiality.

**Figure 16:**
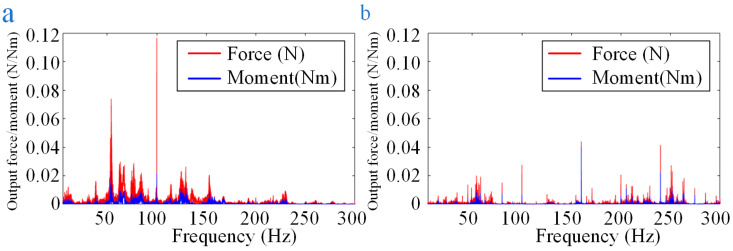
The prediction of interference in one part of the CSST: (**a**) for shutter operation; (**b**) for cryocooler operation.

Figure 16 depicts the prediction of interference in one part of the CSST for shutter operation or cryocooler operation under specific parameter configurations. Their RSM values for combined disturbance forces and moments are within 0.4 N and 0.1 Nm of the operational limits of the telescope, respectively. These results are not affected by the influence of the flexible measuring platform. This provides a valuable reference for the design, control, optimization, and validation of the CSST.

The suggested measuring device has a wide range of potential applications and is not just limited to measuring aerospace precision devices. It can also be used to measure dynamic force in photolithography, medical devices, biological instruments, and other fields. However, there are some limitations to the device that is being presented. For instance, in dynamic force prediction, acquiring the transfer function of complicated and large-scale devices through simulation takes a lot of time and computational power, which significantly extends the project’s cycle time. Nevertheless, it might be possible to increase this efficiency while maintaining its accuracy through proxy modeling and other techniques. In the future, these will be thoroughly examined.

## 6. Conclusions

As telescopes become more accurate and increase in mass and volume, a large dynamic force measuring device is needed to measure and predict the dynamic forces of precision devices with higher accuracy. This paper describes a flexible measuring platform capable of measuring the dynamic disturbance force/moment from large sources considering vibration coupling. Furthermore, a method for predicting the impact of vibration sources on the module is proposed. The flexible measuring platform has greatly increased its load capacity by using three measuring modules and redundant sensors, while also allowing it to install large vibration sources. By considering the vibration coupling between the vibration source, the measuring platform, and the module, both measurement and prediction accuracy are effectively improved. According to the experimental results, the average dynamic measurement error of the flexible measuring platform is within 8% when coupling is considered, which is 29% less when coupling is not considered. In addition, the accuracy error of the prediction model is within 17%. This flexible platform and prediction method have already been applied in practice. Therefore, the platform and methods proposed in this paper can provide a good reference for assessing the effects of vibration sources. In the future, research will concentrate on the extremely challenging problem of online measurement and prediction of the disturbance forces from vibration sources in spacecraft as opposed to the earlier ground-based measurements.

## Figures and Tables

**Table 1:** Parameters of sensors.

No.	Model	Sensitivity	Range	Preload	Resolution
1	9134B, Kistle	−3.8 pC/N	26 kN	15–25 Nm	—
2	208C02, PCB	11.241 mV/N	±2224 N-pk	—	0.004 N-rms

**Table 2:** Material properties during simulation.

Component	*E* (MPa)	*ν*	*ρ* (kg/m^3^)
Installation plate, base, and tooling	2.06 × 10^5^	0.3	7.85 × 10^3^
Sensors (experimental acquisition)	7.65 × 10^4^	0.32	7.5 × 10^3^
Flexible board	7.2 × 10^4^	0.33	2.7 × 10^3^

**Table 3:** Data for key frequencies are shown in Figure 11.

Speed (RPM)			3000	2500	2000	1500	1000	500
Point 1	Black line	Frequency (Hz)	50.25	41.5	33	25.13	16.5	8.5
		Force (N)	1.396	1.175	0.6374	0.3681	0.1884	0.0629
	Red line	Frequency (Hz)	50.62	42.21	33.92	25.69	16.97	8.8
		Force (N)	1.902	1.383	1.672	1.276	0.24	0.0633
	Blue line	Frequency (Hz)	50	41.92	33.08	25.15	16.41	8.33
		Force (N)	1.563	1.194	0.6126	0.3672	0.1705	0.0684
Point 2	Black line	Frequency (Hz)	99.63	83.01	66.63	——	——	——
		Force (N)	0.1518	0.0525	0.0264	——	——	——
	Red line	Frequency (Hz)	101.6	84.67	67.71	——	——	——
		Force (N)	0.1137	0.0462	0.0236	——	——	——
	Blue line	Frequency (Hz)	99.51	83.96	67.04	——	——	——
		Force (N)	0.1554	0.0543	0.0214	——	——	——
Point 3	Black line	Frequency (Hz)	149.8	124.6	——	——	——	——
		Force (N)	0.1696	0.0364	——	——	——	——
	Red line	Frequency (Hz)	152.5	127	——	——	——	——
		Force (N)	0.0766	0.0453	——	——	——	——
	Blue line	Frequency (Hz)	151.3	125.4	——	——	——	——
		Force (N)	0.1845	0.0311	——	——	——	——
Point 4	Black line	Frequency (Hz)	199.9	166.9	——	——	——	——
		Force (N)	0.3514	0.1073	——	——	——	——
	Red line	Frequency (Hz)	202.9	169.5	——	——	——	——
		Force (N)	0.2901	0.1103	——	——	——	——
	Blue line	Frequency (Hz)	200.9	167.5	——	——	——	——
		Force (N)	0.2891	0.0839	——	——	——	——
Point 5	Black line	Frequency (Hz)	250	208.4	——	——	——	——
		Force (N)	0.1405	0.1424	——	——	——	——
	Red line	Frequency (Hz)	253.6	211.9	——	——	——	——
		Force (N)	0.4926	0.0464	——	——	——	——
	Blue line	Frequency (Hz)	251	209.6	——	——	——	——
		Force (N)	0.0755	0.1373	——	——	——	——

## Data Availability

Data are contained within the article.
